# Imaging and blood flow characteristics of cerebrovascular fenestration malformation and its relationship with the occurrence of ischemic cerebrovascular disease

**DOI:** 10.1186/s40001-024-01853-0

**Published:** 2024-05-17

**Authors:** Weifang Xing, Wensheng Zhang, Minzhen Zhu, Yangchun Wen, Yunqiang Huang, JinZhao He

**Affiliations:** Department of Neurology, Heyuan People’s Hospital, Guangdong Provincial People’s Hospital Heyuan Hospital, Heyuan, 517000 Guangdong China

**Keywords:** Cerebrovascular fenestration malformation, Cerebral infarction, Clinical characteristics, Transcranial Doppler cerebral blood flow

## Abstract

**Objective:**

To explore the imaging and transcranial Doppler cerebral blood flow characteristics of cerebrovascular fenestration malformation and its relationship with the occurrence of ischemic cerebrovascular disease.

**Methods:**

A retrospective analysis was conducted on the imaging data of 194 patients with cerebrovascular fenestration malformation who visited the Heyuan People’s Hospital from July 2021 to July 2023. The location and morphology of the fenestration malformation blood vessels as well as the presence of other cerebrovascular diseases were analyzed. Transcranial Doppler cerebral blood flow detection data of patients with cerebral infarction and those with basilar artery fenestration malformation were also analyzed.

**Results:**

A total of 194 patients with cerebral vascular fenestration malformation were found. Among the artery fenestration malformation, basilar artery fenestration was the most common, accounting for 46.08% (94/194). 61 patients (31.44%) had other vascular malformations, 97 patients (50%) had cerebral infarction, of which 30 were cerebral infarction in the fenestrated artery supply area. 28 patients with cerebral infarction in the fenestrated artery supply area received standardized antiplatelet, lipid-lowering and plaque-stabilizing medication treatment. During the follow-up period, these patients did not experience any symptoms of cerebral infarction or transient ischemic attack again. There were no differences in peak systolic flow velocity and end diastolic flow velocity, pulsatility index and resistance index between the ischemic stroke group and the no ischemic stroke group in patients with basal artery fenestration malformation (*P* > 0.05).

**Conclusion:**

Cerebrovascular fenestration malformation is most common in the basilar artery. Cerebrovascular fenestration malformation may also be associated with other cerebrovascular malformations. Standardized antiplatelet and statin lipid-lowering and plaque-stabilizing drugs are suitable for patients with cerebral infarction complicated with fenestration malformation. The relationship between cerebral blood flow changes in basilar artery fenestration malformation and the occurrence of ischemic stroke may not be significant.

## Introduction

Cerebrovascular fenestration malformation refers to the localized duplication of blood vessels that divide into two branches during their course and then merge again into one branch after a certain length. It is a rare congenital vascular developmental anomaly caused by local fusion failure or persistent residual blood vessels during embryonic development, and is often found during autopsy or imaging examinations [1, 2]. At present, there are literature reports that cerebral vascular fenestration abnormalities may be related to the occurrence of cerebral infarction or transient ischemic attacks [3–7]. Therefore, we analyzed the imaging data of 194 patients with cerebrovascular fenestration malformation discovered by electronic computed tomography angiography (CTA), magnetic resonance angiography (MRA) and cerebral digital subtraction angiography (DSA) at the Heyuan People’s Hospital and analyzed the clinical characteristics as well as follow-up situation of 97 patients who suffered from cerebral infarction. Besides, we analyzed the transcranial Doppler cerebral blood flow detection data of patients with basilar artery fenestration malformation in order to improve the understanding of cerebrovascular fenestration malformation and explore the possible relationship between fenestration malformation and cerebral infarction, treatment plan and prognosis of cerebral infarction patients combined with fenestration malformation. This study has passed the ethical review of the Ethics Committee of Heyuan People’s Hospital.

## Materials and methods

### Research object

This study is a retrospective study. We retrospectively analyzed the clinical data of patients who underwent head and neck CTA (Machine types include GE256, GE64 and Siemens 16), MRA (Machine types include GE1.5T and Lianying 3.0T) or DSA (Machine type is Philips) examinations at Heyuan People’s Hospital from July 2021 to July 2023 and analyzed the clinical data of all patients who were found with cerebrovascular fenestration malformation. Inclusion criteria: The imaging characteristics of cerebral vascular fenestration malformation should comply with the manifestation that the blood vessels are divided into two branches during the course of travel and then merge into one branch after a certain length of travel. The imaging data should be interpreted by two attending neurologists and one attending radiologist. If there is a difference of opinion, it should be discussed and decided by both parties. Exclusion criteria: Patients with imaging data that do not meet the criteria of cerebrovascular fenestration malformation. Patients of this research experienced cerebral infarction between July 2021 and July 2023, and we also tracked whether new cerebral infarction occurred again 6–12 months after the stroke.

### Research methods

Detailed records of the patient's general information, the location and morphology of fenestration malformation as well as the presence of other cerebrovascular diseases were recorded. Clinical data of the patient were reviewed and the relationship between the infarct focus and fenestration malformation blood vessels in patients with cerebral infarction was analyzed. The diagnostic criteria for cerebral infarction in this study were: symptoms and signs of focal brain damage and detection of new infarcted lesions on CT or MRI examination. Among young patients, we also conducted examinations for patent foramen ovale, vasculitis and so on, but we had not yet found any rare underlying causes of cerebral infarction in these patients. Patients with basilar artery fenestration malformation were included in the study, and they or their family members had signed an informed consent form to participate in the clinical study. We divided patients with basilar artery fenestration malformation into ischemic stroke group and no ischemic stroke group. Transcranial Doppler cerebral blood flow detection data of patients with basilar artery fenestration malformation were analyzed and the clinical situation of these patients was followed up. The transcranial Doppler ultrasound machine we use is Delicate EMS-9PB. We detect the basilar artery through the occipital window, with a depth of 80–110 mm. Our TCD examination was conducted by the same experienced examiner, who was familiar with the presence of basilar artery window malformations through imaging examinations of the patient before the examination. The examiner repeatedly confirmed increase or decrease in blood flow velocity and the presence of turbulence, eddies, and microemboli and measured PSV/EDV, PI, and RI at a depth of 80–110 mm in the occipital window, which meant detecting and tracking cerebral blood flow throughout the entire basilar artery, so as to clarify whether there are any abnormalities in the cerebral blood flow characteristics of the window opening deformity.

### Statistical methods

Statistical analysis was conducted using SPSS 20.0 statistical software. For metric data that conformed to a normal distribution, the mean ± standard deviation (± S) was used to represent it and the counting data were analyzed using Chi-square test. For count data, the example (%) was used to represent it and the comparison between groups was conducted using independent sample *t*-test. *P* ≤ 0.05 indicated that the difference was considered to be statistically significant.

## Results

1. General information of the patients

From July 2021 to July 2023, more than 40000 patients underwent head and neck CTA, MRA or DSA examinations. The majority of patients undergoing imaging examination were due to considerations of cerebrovascular disease. Among them, 194 patients with cerebrovascular fenestration malformation were found, with a detection rate of approximately 0.49%, including 124 males and 70 females, with a male to female ratio of 1–0.56. The average age of the ischemic stroke group was 65.09 ± 9.42 years old, while the average age of the no ischemic stroke group was 60.80 ± 10.07 years old (see Table 1 for details). Except for cerebrovascular fenestration, other risk factors of stroke mainly include smoking, drinking, hypertension, diabetes, hyperlipidemia and atrial fibrillation.

2. The location and morphology of cerebrovascular fenestration malformation

**Table 1:** General information

Variable	Ischemic stroke group	No ischemic stroke group	Total
Number of cases	97	97	194
Gender (male/female)	69/28 (71.13%/28.87%)	55/42 (56.70%/43.30%)	124/70 (63.92%/36.08%)
Average age (year)	65.09 ± 9.42	60.80 ± 10.07	–
Merge other stroke risk factors (≥ 1) (case)	74 (76.29%)	59 (60.82%)	133 (68.56%)
Receiving secondary preventive treatment for ischemic stroke (Case)	94 (96.91%)	51 (52.58%)	145 (74.74%)
Ischemic stroke after 6 months of follow-up (case)	9 (9.28%)	2 (2.06%)	11 (5.67%)

**Fig. 1:**
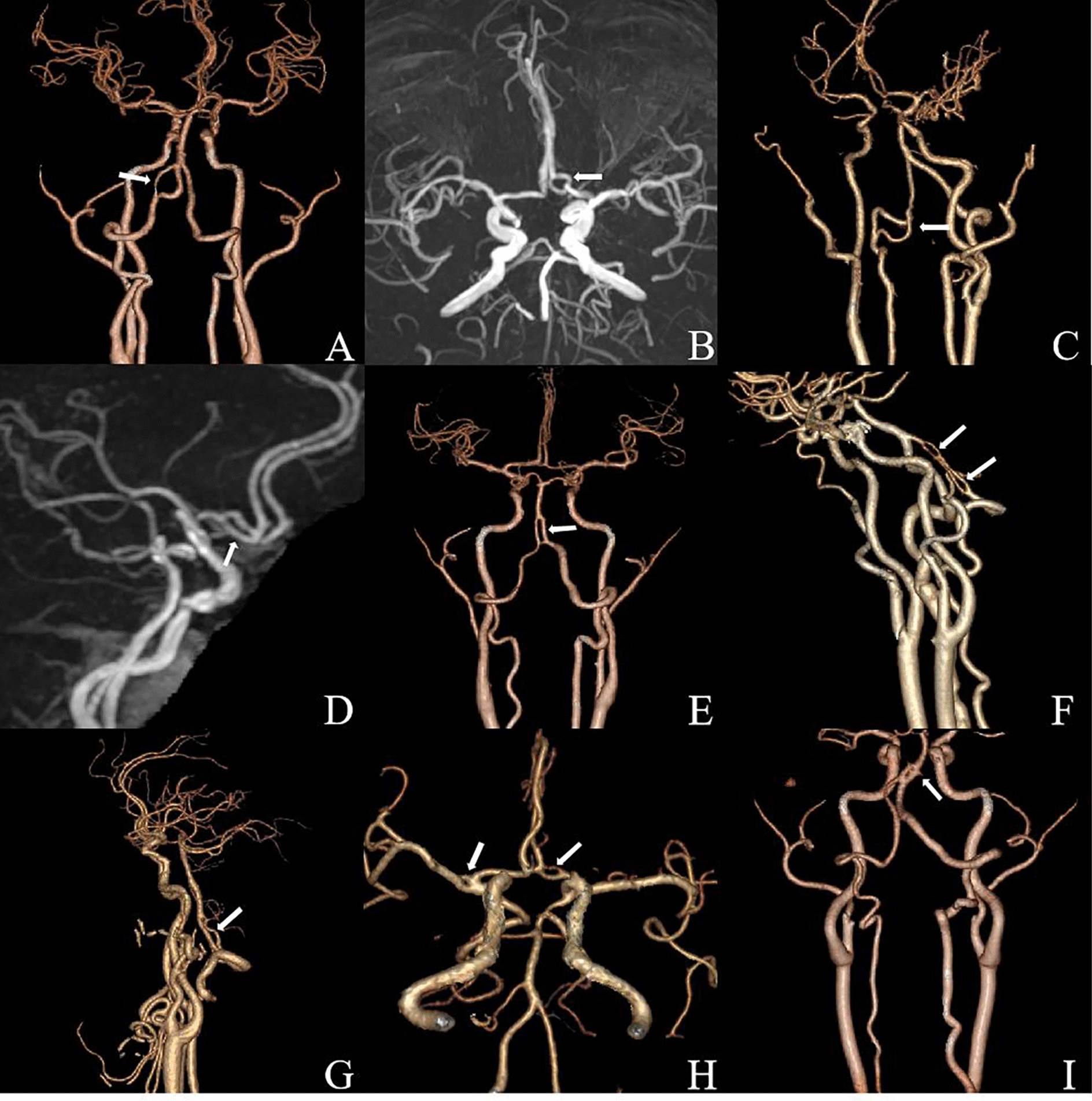
**A**: Left vertebral artery V4 segment convex lens fenestration malformation (arrow). **B**: Left anterior cerebral artery A1 segment convex lens shaped fenestration malformation (arrow). **C**: Parallel repetitive fenestration malformation of the V2–3 segment of the right vertebral artery (arrow). **D**: Left anterior cerebral artery A1 segment parallel repetitive fenestration malformation (arrow). **E**: Parallel repetitive fenestration malformation of the proximal basilar artery (arrow). **F**: Two parallel repetitive fenestration malformation in the V4 segment of the left vertebral artery (arrow). **G**: Irregular fenestration malformation of the intracranial segment of the left vertebral artery (arrow). **H**: Right middle cerebral artery M1 segment slit type fenestration malformation (arrow) combined with left anterior cerebral artery A1 segment convex lens type fenestration malformation (arrow). **I**: 8-shaped fenestration malformation of the proximal basilar artery (arrow)

A total of 204 vascular fenestration abnormalities were found in 194 patients. Among them, 94 cases (46.08%) had fenestration malformation in the basilar artery, 36 cases (17.65%) in the vertebral artery, 47 cases (23.04%) in the anterior cerebral artery, 15 cases (7.35%) in the anterior communicating artery, 10 cases (4.90%) in the middle cerebral artery, 1 case (0.49%) in the posterior cerebral artery and 1 case (0.49%) in the internal carotid artery. The morphology of fenestrated blood vessels was divided into fissure type, convex lens type, parallel duplication type and other types, with 106 cases of fissure type (53.27%), 43 cases of convex lens type (21.61%), 47 cases of parallel duplication type (23.62%) and 3 cases of other types (including double duplication type, irregular type and 8-shaped type) (1.51%). The images of some cerebrovascular fenestration malformation are shown in Fig. 1.

3. Cerebrovascular fenestration malformation combined with other cerebrovascular diseases

Out of 194 patients with cerebrovascular fenestration malformation, 61 (31.44%) had other cerebrovascular malformations. Among them, there were 38 cases of combined embryonic posterior cerebral artery, 12 cases of missing A1 segment of anterior cerebral artery, 2 cases of aneurysm, 2 cases of vagal vertebral artery, 5 cases of bilateral anterior cerebral artery trunk and 10 cases of other cerebrovascular malformations (including moyamoya disease, moyamoya syndrome, etc.).

4. The situation of patients with cerebrovascular fenestration malformation combined with cerebral infarction

Among the 194 patients, 97 cases were complicated with cerebral infarction, of which 30 cases (30.93%) were cerebral infarction in the fenestrated artery supply area. The severity of their cerebral infarction varies, with symptoms including dizziness, limb weakness, numbness, and unclear speech and so on depending on the affected cerebral blood vessels. 28 patients (93.33%) with cerebral infarction in the fenestrated artery supply area received long-term and standardized secondary prevention therapy mainly including antiplatelet and statin lipid-lowering and plaque-stabilizing drugs. After 6–12 months of follow-up, all patients with a history of cerebral infarction in the fenestrated artery supply area and receiving long-term and standardized secondary prevention therapy did not experience any new symptoms of cerebral infarction or transient ischemic attack and their NIHSS (National Institute of Health stroke scale) and mRS (modified Rankin Scale) were both 0 point.

5. Characteristics of transcranial Doppler cerebral blood flow

Ischemic stroke group and no ischemic stroke group that with basilar artery fenestration malformation included 25 and 10 cases, respectively, with 6 cases of increased PI value, 7 cases of decreased PI value, and 22 cases of normal range. Statistical analysis was conducted on the transcranial Doppler cerebral blood flow detection data of all patients with basilar artery fenestration malformation. The analysis items included peak systolic flow velocity (PSV), end diastolic flow velocity (EDV), pulsatility index (PI), and resistance index (RI), with *P* values greater than 0.05 (see Table 2 for details), indicating that there was no differences in PSV, EDV, PI and RI between the two groups. Only one patient with basilar artery fenestration malformation with posterior circulation cerebral infarction was found to have eddy currents in the basilar artery through transcranial Doppler cerebrovascular examination.

**Table 2:** Characteristics of transcranial Doppler cerebral blood flow in patients with basilar artery fenestration malformation

Variable	Ischemic stroke group (*n* = 25)	No ischemic stroke group (*n* = 10)	*t* value	*P* value
PSV (cm/s)	60.7 ± 20.75	57.6 ± 20.52	0.542	0.591
EDV (cm/s)	22.2 ± 5.12	27.3 ± 8.86	− 0.539	0.594
PI	1.06 ± 0.47	0.80 ± 0.24	1.101	0.279
RI	0.60 ± 0.14	0.51 ± 0.10	1.162	0.254
Number of cases of turbulence or eddy current in blood vessels	1	0	/	/

*PSV* Peak systolic flow velocity, *EDV* end diastolic flow velocity, *PI* pulsatility index, *RI* resistance index

*P* < 0.05 indicates a statistically significant difference between the two groups

## Discussion

Cerebrovascular fenestration malformation is a rare congenital vascular developmental anomaly that is closely related to the process of embryonic formation. The current research reported an incidence rate of approximately 4.6% for cerebrovascular fenestration malformation. In this study, cerebrovascular fenestration specifically referred to cerebral artery fenestration, and venous fenestration was not included. The most common blood vessel for cerebral arterial fenestration malformations in our study was the basilar artery, with an incidence rate of 1.37–2.33%, accounting for 32.1–42.1% of all cerebrovascular fenestration malformations. Other cerebral blood vessels included the vertebral artery accounting for 24.0–26.3%, the middle cerebral artery accounting for 19.3–23.7% and the anterior cerebral artery accounting for 7.9–17.2% [8–11]. The detection rate of cerebrovascular fenestration malformation in this study was 0.49%, which is lower than the detection rate of previous studies. The detection rate of basilar artery fenestration malformation in this study was only 0.24%, accounting for 46.08% of all fenestration malformation in this study. The detection rate of cerebrovascular fenestration malformation in this study was relatively low, possibly due to the low incidence of cerebrovascular fenestration malformation in the local area, or the fact that many healthy individuals with concomitant cerebrovascular fenestration malformations had not undergone cerebral vascular examination. In addition, this study did not include cases of venous fenestration [12], which may be one of the reasons for the low detection rate of vascular fenestration malformation.

According to the morphology of cerebrovascular fenestration malformation, previous studies had mainly classified cerebrovascular fenestration malformation into three types: slit type, convex lens type and parallel duplication type. Among them, the characteristic of fissure type fenestration malformation is short blood vessels and small blood vessel gaps; the characteristic of convex lens type fenestration deformity is that the fenestration vessels are slightly larger and the vascular gaps are obvious; the characteristic of parallel repetitive fenestration malformation is the complete duplication of arteries. Our study found that fissure type cerebrovascular fenestration malformation is the most common which is similar to previous studies [12]. In our study, we found that some vascular fenestration malformation cannot be classified as slit type, convex lens type or parallel duplication type, such as double duplication, irregular type, 8-shaped type, etc. We temporarily classified these cerebrovascular fenestration malformations as other types.

Previous studies had found that cerebrovascular fenestration malformation is often associated with other cerebrovascular diseases, such as aneurysms, moyamoya disease, venous malformations, etc. [4–7], which also suggests that cerebrovascular fenestration malformation is related to congenital developmental abnormalities. Our study found that embryonic posterior cerebral artery is the most common type of other cerebrovascular diseases combined with cerebrovascular fenestration malformation.

In recent years, there have been reports and studies on cases of cerebrovascular fenestration malformation combined with ischemic stroke [13–15]. However, the pathogenesis of ischemic stroke related to cerebrovascular fenestration malformation is still unclear. Jeong et al. [3] reported 5 cases of cerebral infarction patients with middle cerebral artery fenestration malformation and analyzed that the mechanism of fenestration artery thrombosis may be due to hemodynamic changes at the fenestration artery branch inducing thrombosis. In addition, they found that in two asymmetric blood vessels where fenestration is performed, the smaller branch may be more susceptible to being affected and be more likely to form low shear forces, eddies or turbulence due to the formation of branches, forming microthrombosis and subsequently embolizing the corresponding cerebral vascular supply area. We analyzed the transcranial Doppler cerebral blood flow data of 35 patients with basilar artery fenestration malformation and found that there were no differences in PSV, EDV, PI and RI of the basilar artery between the ischemic stroke group and the no ischemic stroke group. In addition, only one patient with basilar artery fenestration malformation combined with posterior circulation cerebral infarction experienced eddy current in the examination of transcranial Doppler cerebral blood flow. Our study found that the cerebral blood flow characteristics of basilar artery fenestration malformation may not be closely related to the occurrence of ischemic stroke which is different from the report by Jeong et al. If patients with cerebral vascular fenestration malformation suffer from cerebral infarction, but they are combined with moyamoya disease or moyamoya syndrome, the impact of moyamoya disease and moyamoya syndrome on the occurrence of cerebral infarction may be greater than that of cerebral vascular fenestration malformation. However, the number of cases included in our study was relatively limited. Next, a larger sample size and clinical data are needed to confirm the relationship between cerebrovascular fenestration malformation and the occurrence of ischemic stroke.

Our study fully utilized transcranial Doppler ultrasound to perform cerebral blood flow examination of the cerebrovascular fenestration malformation. To our best knowledge, our study is the first to explore the characteristics of cerebral blood flow in cerebrovascular fenestration malformation. We dynamically observed the blood flow of blood vessels in fenestration malformation, clarified whether there are changes in blood flow velocity that increase or decrease, whether eddies occur, whether turbulence occurs and whether arteriosclerosis is merged. In addition, our study also conducted an observational study on secondary prevention with antiplatelet, lipid-lowering and plaque-stabilizing drugs in patients with cerebrovascular fenestration malformation who may develop new ischemic stroke. It was found that standardized secondary prevention can effectively prevent the occurrence of acute ischemic stroke for the patients with cerebrovascular fenestration malformation, which is also an innovation of this study.

In summary, cerebrovascular fenestration malformation is a congenital anomaly of cerebral vascular development, most commonly occurring in the basilar artery, and may be accompanied by other vascular malformation such as cerebral aneurysms, moyamoya disease and so on. The relationship between cerebral blood flow changes in basilar artery fenestration malformation and the occurrence of ischemic stroke may not be significant. Long-term and standardized secondary prevention measures such as antiplatelet and statin lipid-lowering and plaque-stabilizing drugs are effective for cerebral infarction patients with cerebrovascular fenestration malformation.

## Data Availability

The datasets analyzed during the current study are not publicly available due to intellectual property rights, but are available from the corresponding author on reasonable request.

## Abbreviations

CTA: Computed tomography angiography
MRA: Magnetic resonance angiography
DSA: Digital subtraction angiography
PSV: Peak systolic flow velocity
EDV: End diastolic flow velocity
PI: Pulsatility index
RI: Resistance index
NIHSS: National Institute of Health stroke scale
mRS: Modified Rankin Scale
